# Screening of postpartum diabetes in women with gestational diabetes: high-risk subgroups and areas for improvements—the STRONG observational study

**DOI:** 10.1007/s00592-021-01707-9

**Published:** 2021-04-12

**Authors:** Angela Napoli, Laura Sciacca, Basilio Pintaudi, Andrea Tumminia, Maria Grazia Dalfrà, Camilla Festa, Gloria Formoso, Raffaella Fresa, Giusi Graziano, Cristina Lencioni, Antonio Nicolucci, Maria Chiara Rossi, Elena Succurro, Maria Angela Sculli, Marina Scavini, Ester Vitacolonna, Matteo Bonomo, Elisabetta Torlone, the STRONG Study Collaborators and the AMD-SID Diabetes and Pregnancy Study Group, Angela Napoli, Olimpia Bitterman, Camilla Festa, Chiara Giuliani, Matteo Bonomo, Basilio Pintaudi, Elena Cimino, Elena Mion, Teresa Marcone, Cristina Lencioni, Graziano Di Cianni, Laura Sciacca, Andrea Tumminia, Agostino Milluzzo, Ester Vitacolonna, Federica Fraticelli, Marica Franzago, Alessandro Roberto Dodesini, Elena Ciriello, Mariagrazia Dalfrà, Annunziata Lapolla, Raffaella Fresa, Aurora Grassi, Paolo Limone, Annamaria Nuzzi, Andi Masha, Laura Grimaldi, Sara Biglino, Egle Ansaldi, Maurizia Battezzati, Giancarla Meregalli, Valentina De Mori, Denise Berzi, Antonio Bossi, Viviana Baggi, Elisabetta Lovati, Lara Quarleri, Tiziana Romanelli, Silvia Clementi, Ilaria Nicolao, Francesca Zambotti, Simonetta Lombardi, Silvana Costa, Chiara Tommasi, Silvia Rancan, Giovanna Lisato, Paola Bordon, Daniela Turazzi, Francesco Mollo, Franco Grimaldi, Laura Tonutti, Sandra Agus, Maria Rosaria Falivene, Giorgio Versari, Laura Corsi, Maria Delucchi, Luisa Ratto, Maria Grazia Magotti, Tiziana Frusca, Silvia Haddoub, Alice Suprani, Mary Mori, Maria Grazia Vita, Nicolina Di Biase, Alessandra Bertolotto, Michele Aragona, Cristina Bianchi, Emilia Lacaria, Elisa Guarino, Federica Monaci, Francesco Dotta, Elisabetta Torlone, Carlo Lalli, Chiara Di Loreto, Maura Scarponi, Angela Del Prete, Sergio Leotta, Iolanda Coletta, Santina Abbruzzese, Valeria Montani, Emanuela Cannarsa, Pierpaolo Contini, Raffaella Vero, Rosa Oliverio, Marina Scavini, Nicoletta Dozio, Maria Pia Imbergamo, Renzo Cordera, Laura Affinito, Davide Maggi, Caterina Bordone, Elena Fochesato, Alessandra Pissarelli, Eros Libera, Susanna Morano, Tiziana Filardi, Mara Fallarino

**Affiliations:** 1 AMD-SID Diabetes and Pregnancy Study Group, Rome, Italy; 2 Department of Clinical and Molecular Medicine, Sant’Andrea Hospital, Faculty of Medicine and Psychology, Sapienza University, Rome, Italy; 3 Department of Clinical and Experimental Medicine, Endocrinology Section, University of Catania Medical School, Catania, Italy; 4 SSD Diabetology, Ca’Granda Niguarda Hospital, Milan, Italy; 5 Department of Medicine, University of Padova, Padova, Italy; 6 Department of Medicine and Aging Sciences; Center for Advanced Studies and Technology (CAST, Ex CeSI-Met), G. D’Annunzio University, Chieti, Italy; 7 Endocrinology and Diabetes Unit, ASL Salerno, Salerno, Italy; 8 CORESEARCH – Center for Outcomes Research and Clinical Epidemiology, Pescara, Italy; 9 Diabetes and Endocrinology Unit, Usl Nord Ovest Tuscany, Lucca, Italy; 10 Department of Medical and Surgical Sciences, University Magna Graecia of Catanzaro, Catanzaro, Italy; 11 Endocrinology and Diabetes, Bianchi Melacrino Morelli Hospital, Reggio Calabria, Italy; 12 Division of Immunology, Transplantation and Infectious Diseases, Diabetes Research Institute (DRI), IRCCS San Raffaele Scientific Institute, Milan, Italy; 13 Department of Medicine and Aging, School of Medicine and Health Sciences, “G. D’Annunzio” University, Chieti-Pescara, Chieti, Italy; 14 Internal Medicine, Endocrinology and Metabolism, S. Maria Della Misericordia Hospital, Perugia, Italy

**Keywords:** Gestational diabetes, Postpartum dysglycemia, Risk factors, Type 2 diabetes, Obesity

## Abstract

**Aims:**

To assess the proportion of women with gestational diabetes (GDM) by performing postpartum Oral Glucose Tolerance Test (OGTT) and to identify GDM phenotypes at high-risk of postpartum dysglycemia (PPD).

**Methods:**

Observational, retrospective, multicenter study involving consecutive GDM women. Recursive partitioning (RECPAM) analysis was used to identify distinct and homogeneous subgroups of women at different PPD risk.

**Results:**

From a sample of 2,736 women, OGTT was performed in 941 (34.4%) women, of whom 217 (23.0%) developed PPD. Insulin-treated women having family history of diabetes represented the subgroup with the highest PPD risk (OR 5.57, 95% CI 3.60–8.63) compared to the reference class (women on diet with pre-pregnancy BMI <  = 28.1 kg/m^2^). Insulin-treated women without family diabetes history and women on diet with pre-pregnancy BMI > 28.1 kg/m^2^ showed a two-fold PPD risk. Previous GDM and socioeconomic status represent additional predictors. Fasting more than post-prandial glycemia plays a predictive role, with values of 81–87 mg/dl (4.5–4.8 mmol/l) (lower than the current diagnostic GDM threshold) being associated with PPD risk.

**Conclusions:**

Increasing compliance to postpartum OGTT to prevent/delay PPD is a priority. Easily available characteristics identify subgroups of women more likely to benefit from preventive strategies. Fasting BG values during pregnancy lower than those usually considered deserve attention.

## Introduction

Gestational diabetes mellitus (GDM) represents a pathological condition for the mother and the fetus during pregnancy, at delivery and in the follow-up period [1]. Prevalence varies in the different countries and in Italy it is estimated that 11% of pregnancies are aggravated by GDM [2, 3].

Women with GDM have an increased risk of adverse obstetric events and adverse neonatal outcomes compared to women with physiological pregnancy, including fetal macrosomia, shoulder dystocia, neonatal trauma, neonatal jaundice, respiratory distress, and neonatal hypoglycemia [4–6].

GDM is also associated with an increased risk of dysglycemia and type 2 diabetes development after delivery compared to normal pregnancy [7, 8]. Women with gestational diabetes have a 7–12 times higher risk of developing type 2 diabetes compared to women with normoglycemic pregnancy [7].

Recent evidence underlines the importance of early identification of GDM and its subsequent treatment to promote maternal–fetal health [9]. Based on the HAPO (Hyperglycemia and Adverse Pregnancy Outcomes) study, the IADPSG panel defined new guidelines for GDM, improving criteria for screening, diagnosis and treatment of GDM [10]. In Italy, the ‘Guideline on physiological pregnancy’’ was also developed, to disseminate specific recommendation on GDM, describing screening and diagnostic procedures. A selective screening, based on the presence of specific risk factors, is recommended after the exclusion of overt diabetes; this guideline also recommends a follow-up OGTT to be performed not before 6 weeks from the delivery [11, 12].

A previous publication based on the STRONG study aimed to assess the risk of adverse neonatal outcomes in women with GDM and to identify subgroups of women at high risk for adverse neonatal outcomes [13]. This secondary analysis, based on the same data, aimed at evaluating compliance with follow-up OGTT and identifying subgroups of women at high risk for developing postpartum dysglycemia (PPD).

## Methods

This was an observational, retrospective, multicenter study. Details on materials and methods of the study protocol have been published elsewhere [13].

The study involved consecutive women with pregnancy complicated by GDM cared for by a network of Italian outpatient diabetes clinics.

Women with the following characteristics were eligible for this study: age ≥ 18 years, GDM, delivery by the end of the planned study period (May 2015), signature of informed consent.

Exclusion criteria were: diagnosis of pre-gestational diabetes, twin pregnancy.

According to current Italian recommendations [11, 12], all women with a physiologic pregnancy receive an evaluation of fasting plasma glucose during the first trimester. Women with at least one risk factor (age >  = 35 years, first-degree relative with diabetes, BMI > 25 kg/m^2^, Asian, Middle Eastern, or Caribbean ethnicity) perform the screening for GDM at 24–28th weeks of gestation. Nevertheless, it is recommended that women with at least one of the following conditions should be screened at 16–18 weeks of gestation: previous GDM, pre-pregnancy body mass index (BMI) ≥ 30 kg/m^2^, plasma glucose values at the beginning of pregnancy (within the first trimester) between 100 and 125 mg/dl (5.6–6.9 mmol/l).

An oral glucose tolerance test with 75 g of glucose (OGTT-75 g) is performed.

Women with one or more plasma glucose values above the established thresholds (≥ 92 mg/dl/5.1 mmol/l at baseline (T0′), ≥ 180 mg/dl/10.0 mmol/l after 1 h (T60′) from the load, ≥ 153 mg/dl/8.5 mmol/l after 2 h (T120′) from the load) are diagnosed as affected by GDM. After the diagnosis, according to usual care, women with GDM are invited to perform self-monitoring of blood glucose, to follow a balanced diet, and to do regular physical activity. If blood glucose (BG) is not in target, a pharmacological therapy (in Italy the only approved treatment is insulin) is started.

Follow-up OGTT is recommended within 12 weeks after delivery. Based on follow-up OGTT results, dysglycemia was defined as Impaired Fasting Glucose (IFG, BG levels between 100 and 125 mg/dl/5.6 and 6.9 mmol/l at T0′), Impaired Glucose Tolerance (IGT, BG levels between 140 and 199 mg/dl/7.8 and 11.1 mmol/l at T120′), or type 2 diabetes (BG levels >  = 126 mg/dl/7.0 mmol/l at T0′ or >  = 200 mg/dl/11.1 at T120′).

Data collected included sociodemographic information, clinical characteristics related to the period before and during pregnancy, and information on maternal–fetal outcomes (i.e., adequate, large or small fetal growth for gestational age, macrosomia, minor and major malformations, neonatal intensive care need, neonatal hypoglycemia needing i.v. treatment, neonatal hypocalcemia, neonatal hyperbilirubinemia, shoulder distocia, respiratory distress, type of delivery, stillbirths, maternal mortality, neonatal mortality) [13].

All information was collected on electronic case report forms and data were anonymous.

The study was conducted in accordance with the Helsinki Declaration on Medical Research on Humans and with the Good Clinical Practice (GCP). The study was approved by the Ethics Committees of all participating Centers. Participant patients gave informed consent before taking part in the study.

### Statistical analysis

Descriptive data were summarized as mean and standard deviation or percentages. Characteristics of the study population were assessed overall and by the development of dysglycemia after the delivery.

Groups were compared using Student’s test (continuous, normally distributed variables), Mann–Whitney U-test (continuous, not normally distributed variables), or chi-square test (categorical variables).

RECursive Partitioning and AMalgamation (RECPAM) analysis, a tree-based statistical method that integrates standard regression and tree-growing techniques, was used to detect potential interactions among the different variables in predicting development of postpartum dysglycemia and identify homogeneous and distinct subgroups of patients with increased likelihood of reaching the endpoint [14]. At each partitioning step, the RECPAM method automatically choses the covariate and its best binary split to maximize the difference in risk of experiencing the outcome. The algorithm stops when user-defined stopping rules are met. In this case, each final class was required to have at least 100 patients in total and 30 patients with the target endpoint. The set of variables tested in the RECPAM analysis included: age, ethnicity, education level, occupation, pre-pregnancy BMI, previous GDM, family history (first-degree relatives) of diabetes, physical activity before pregnancy, first pregnancy, smoke and GDM treatment. Continuous variables were not categorized so as to allow the algorithm to choose the natural cut-off points when identifying distinct subgroups of patients. For each subgroup or class, the proportion (%) of patients reaching the endpoint and the likelihood (OR and 95% CI) to reach the endpoint versus the reference subgroup were obtained.

Additional RECPAM analyses were performed to test the role of fasting BG at first trimester of pregnancy and BG values at T0′, T60′, and T120′ of diagnostic OGTT (at 16–18 or 24–28 weeks, separately) in the subgroups of women with available values.

All analyses were performed using the SAS version9.3 (SAS Institute Inc.) program.

## Results

From a total sample of 2,736 women, postpartum OGTT was performed within 6–12 weeks from the delivery in 941 (34.4%) women. Median proportion of women with a follow-up OGTT largely varied among the 42 participating centers (median 42.0%, interquartile range 22.0–80.0%).

Compared to women not performing follow-up OGTT, those performing the test were less often Caucasian, were less likely to be at their first pregnancy, had more frequently had a previous GDM, had lower levels of diastolic blood pressure, total cholesterol and triglycerides, and a more frequent use of insulin for their GDM (Table 1).

**Table 1:** Sociodemographic and clinical characteristics of patients, overall, by availability of follow-up OGTT within 12 weeks from the delivery and by development of postpartum dysglycemia

	Women without follow-up OGTT	Women with follow-up OGTT	*p *value*	No postpartum dysglycemia	Postpartum dysglycemia	*p *value*
*N*	1795	941		724	217	
Age (years)	36.5 ± 5.2	36.7 ± 4.8	0.51	36.8 ± 4.7	36.6 ± 5.4	0.62
School education (%)						
Low	26.3	25.5	0.41	22.6	35.3	**0.002**
Median	47.3	50.6		50.9	49.6	
High	26.3	23.9		26.4	15.1	
Occupation (%)			0.12			
Housewife	36.8	33.2		28.7	48.2	** < 0.0001**
Employed	62.8	65.7		70.1	51.1	
Student	0.4	1.1		1.3	0.7	
Physical activity before pregnancy (%)	24.6	26	0.52	28.8	16.7	**0.002**
Smoke (%)			0.27			
No	77.4	80.2		80	81.1	0.06
Yes	9.9	8.9		8	11.7	
Ex	12.6	10.9		12	7.3	
Caucasian ethnicity (%)	48.5	37.8	** < 0.0001**	36.2	42.9	0.08
Family history of diabetes (%)	41.3	42.4	0.58	38.8	54.7	** < 0.0001**
First pregnancy (%)	47.5	41.1	**0.002**	43.9	31.9	**0.002**
Previous GDM (%)	12.3	17.3	**0.0004**	14.8	25.7	**0.0002**
Pre-pregnancy BMI (kg/m^2^)	26.0 ± 6.0	26.0 ± 5.5	0.57	25.6 ± 5.5	27.4 ± 5.1	** < 0.0001**
Pre-pregnancy BMI in classes (%)						
< 25	52.1	51.0	0.10	55.6	35.4	**< 0.0001**
25–30	26.5	30.0		27.9	37.4	
> 30	21.4	19.0		16.5	27.2	
Weight gain (kg)	9.8 ± 5.9	9.9 ± 5.3	0.98	10.2 ± 4.9	8.8 ± 6.2	**0.0002**
Blood glucose at first trimester (mg/dl)	88.9 ± 11.7	88.7 ± 11.4	0.86	86.8 ± 10.5	94.6 ± 12.1	** < 0.0001**
Diagnostic OGTT (%)						
16–17th week	12.4	16.3	0.02	12.2	32.6	**< 0.0001**
18–24th week	87.6	83.7		87.8	67.4	
HbA1c at diagnosis % (mmol/mol)	32.7 ± 8.3	32.7 ± 8.6	0.29	32.3 ± 7.9	34.0 ± 10.5	** < 0.0001**
Systolic blood pressure (mmHg)	112.7 ± 13.8	111.2 ± 12.8	0.06	111.3 ± 12.8	111.1 ± 12.7	0.75
Diastolic blood pressure (mmHg)	71.0 ± 9.9	69.2 ± 9.2	** < 0.0001**	69.4 ± 9.2	68.7 ± 9.0	0.30
Total cholesterol (mg/dl)	254.5 ± 51.2	247.0 ± 47.8	**0.04**	248.1 ± 47.1	242.6 ± 50.6	0.33
HDL cholesterol (mg/dl)	68.1 ± 15.1	69.3 ± 15.8	0.32	69.5 ± 15.9	68.1 ± 15.4	0.52
LDL cholesterol (mg/dl)	142.0 ± 45.6	142.8 ± 44.1	0.74	143.9 ± 42.1	137.6 ± 52.5	0.14
Triglycerides (mg/dl)	220.6 ± 102.3	186.6 ± 83.1	** < 0.0001**	183.3 ± 79.7	199.8 ± 94.8	0.21
Glucose lowering treatment (%)						
Diet	63.2	50.5	** < 0.0001**	56.3	31.5	** < 0.0001**
Insulin	36.8	49.5		43.7	68.5	
Gestational week at delivery	38.4 ± 1.7	38.6 ± 1.4	**0.03**	38.7 ± 1.4	38.3 ± 1.5	** < 0.0001**
Adverse neonatal outcome (%)	30.3	29.1	0.52	27	36.1	**0.009**

*p values < 0.05 are in bold and expresses statistically significant between-group differences. Student’s test (for continuous, normally distributed variables), Mann–Whitney test (for continuous, not normally distributed variables) or Chi-square test (for categorical variables)

**Fig. 1:**
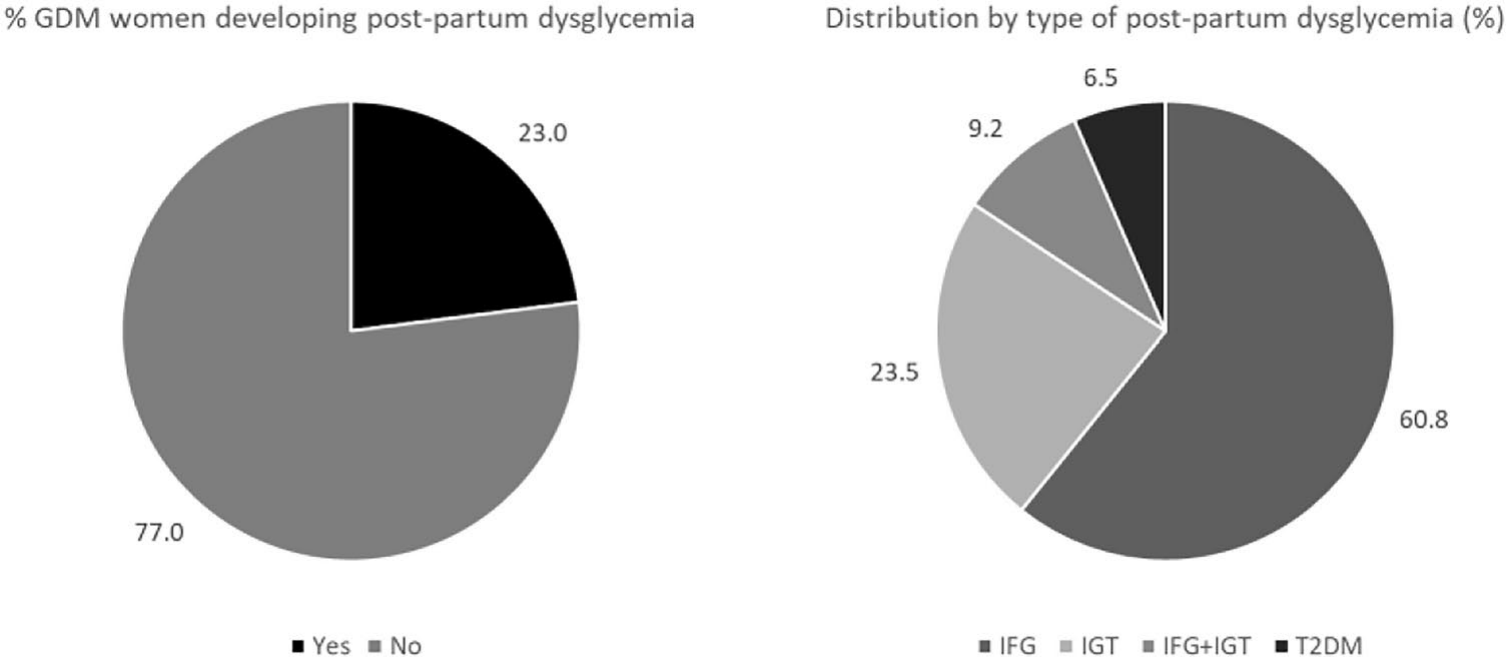
Distribution by development and type of postpartum dysglycemia. *GDM* gestational diabetes mellitus, *IFG* impaired fasting glucose, *IGT* impaired glucose tolerance, *T2DM* type 2 diabetes

Among women performing follow-up OGTT, 217 (23.0%) developed postpartum dysglycemia (Table 1). In particular, 132 (60.8%) women developed IFG, 51 (23.5%) developed IGT, 20 (9.2%) developed both IFG and IGT, and 14 (6.5%) developed type 2 diabetes (Fig. 1).

Compared to women not developing dysglycemia, those developing the condition were more likely to report a low level of school education and being not employed, and less likely to practice physical activity. Pre-pregnancy BMI was significantly higher in women developing dysglycemia, although their weight gain during pregnancy was significantly lower according to IOM guidelines [15]. Furthermore, they more frequently reported family history of diabetes and history of previous GDM. In these women HbA1c levels and BG levels during pregnancy were higher, a higher proportion performed the diagnostic OGTT at 16-18^th^ weeks, and they were treated more often with insulin for GDM. Adverse neonatal outcomes had occurred more frequently in women with postpartum dysglycemia.

**Fig. 2:**
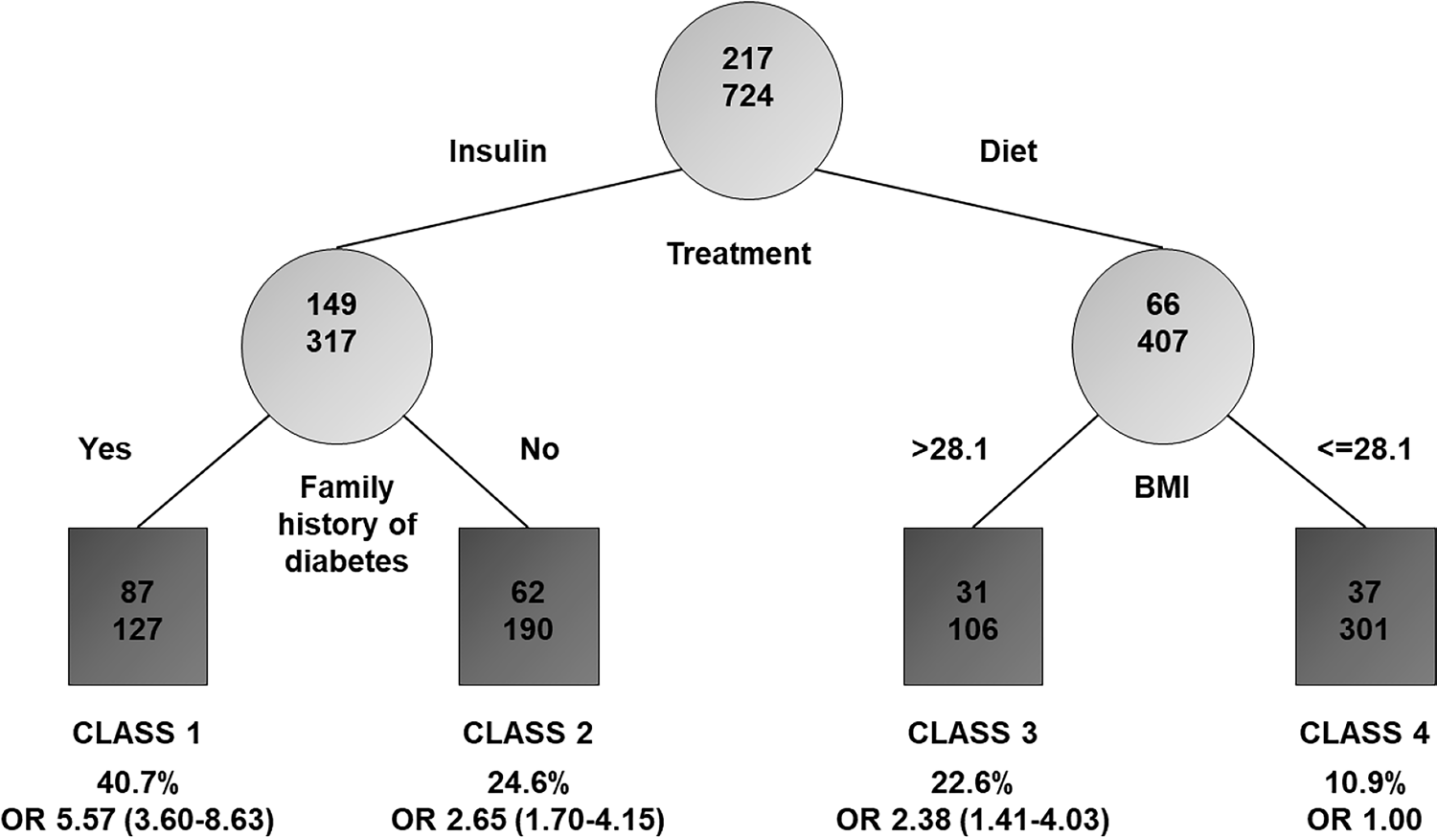
Results of the RECPAM analysis: identification of subgroups at different risks of developing postpartum dysglycemia. The tree-growing algorithm modeled odds ratios (ORs) following a logistic regression model with age, education, occupation, ethnicity, previous GDM, pre-pregnancy BMI, family history of diabetes, first pregnancy, physical activity before pregnancy, smoke, treatment, total cholesterol, HDL, LDL, triglycerides, systolic blood pressure, diastolic blood pressure and composite of adverse neonatal outcomes as covariates. Splitting variables are shown between branches, whereas the condition sending patients to the left or right sibling is on the relative branch. Circles indicate subgroups of patients, squares indicate the final RECPAM classes. Numbers inside circles and squares represent the number of events (top) and the number of nonevents (bottom), respectively. An OR with the corresponding 95% CI (in parentheses) is shown for each class. Class 4, with the lowest risk of developing dysglycemia, is placed at the extreme right and is the reference category (OR = 1)

RECPAM analysis led to the identification of four classes at different risks of developing postpartum dysglycemia (Fig. 2). The most important variables differentiating the risk of developing postpartum dysglycemia were insulin GDM treatment and pre-pregnancy BMI, with patients treated with diet and pre-pregnancy BMI levels <  = 28.1 kg/m^2^ having the lowest prevalence (10.9%). Therefore, this group served as the reference category.

On the opposite side of the regression tree, patients treated with insulin and having family history of diabetes represented the subgroup with the highest prevalence (40.7%) and the highest risk of dysglycemia (OR = 5.57; 95%CI 3.60–8.63).

Women treated with insulin for GDM and no family history of diabetes (class 2) also had a significant risk of adverse outcome compared with the reference category (OR = 2.65; 95% CI 1.70–4.15); similarly, women not treated with insulin and with pre-pregnancy BMI levels > 28.1 kg/m^2^ (class 3) showed a twofold increased risk of dysglycemia (OR = 2.38; 95% CI 1.41–4.03).

The four RECPAM classes differed for other characteristics (Table 2): women with the highest risk of dysglycemia were more likely to have a low level of school education. Average pre-pregnancy BMI substantially differed among classes, ranging from 22.1 to 30.4 kg/m^2^. Results of OGTT performed during pregnancy also varied among classes. Relevantly, in class 4 OGTT test at 16–18 weeks showed levels of BG at T0′ and T60′ markedly lower than the other 3 classes. The four classes also had different levels of systolic and diastolic blood pressure, without a clear trend. Triglycerides mean levels were of 212 mg/dl in class 1, 186 mg/dl in class 2 and 3, and 141 in class 4 (*p* < 0.0001). Levels of follow-up OGTT at T0′ and T120′ decreased from class 1 to class 4. From class 1 to class 4, adverse neonatal outcomes occurred in 37.9%, 31.0%, 24.8%, and 23.8%, respectively (*p* = 0.003).

**Table 2:** Sociodemographic and clinical characteristics of patients by RECPAM classes

	CLASS1	CLASS2	CLASS3	CLASS4	*p* value*
*N*	214	252	137	338	
Age (years)	37.0 ± 5.4	36.5 ± 4.5	37.0 ± 5.1	36.6 ± 4.6	0.59
Education (%)					
Low	36.2	28.3	29.2	16.2	**0.001**
Median	46.5	47.6	51.7	54.7	
High	17.3	24.1	19.1	29.1	
Occupation (%)					
Housewife	39.2	38.9	33.7	25.5	0.06
Employed	60.8	59.9	65.2	72.7	
Student	0	1.2	1.1	1.7	
Physical activity before pregnancy (%)	19.9	22.3	20.2	34.4	**0.002**
Smoke (%)					
No	75.6	78.1	83.8	83.2	**0.01**
Yes	15.0	9.9	6.9	5.1	
Ex	9.3	12.0	9.2	11.7	
Ethnicity (%)					
Caucasian	48.8	36.1	37.5	32.2	**0.002**
Other	51.2	63.9	62.5	67.8	
Family history of diabetes (%)	100	0	41.0	38.6	** < 0.0001**
First pregnancy (%)	26.9	39.7	37.2	52.7	** < 0.0001**
Previous GDM (%)	19.9	22.1	16.9	12.3	**0.01**
Pre-pregnancy BMI (kg/m^2^)	27.8 ± 5.8	27.0 ± 5.7	30.4 ± 4.1	22.1 ± 2.3	** < 0.0001**
Weight gain (kg)	9.8 ± 5.7	10.1 ± 5.6	7.7 ± 5.7	10.7 ± 4.2	** < 0.0001**
Blood glucose at first trimester (mg/dl)	92.2 ± 11.3	91.0 ± 11.8	87.4 ± 8.5	85.1 ± 11.1	** < 0.0001**
HbA1c at diagnosis (mmol/mol)	34.5 ± 8.9	34.6 ± 6.6	32.5 ± 8.1	30.2 ± 9.2	** < 0.0001**
Diagnostic OGTT (%)					
16–17th week	25.0	22.7	17.0	7.1	**< 0.0001**
18–24th week	75.0	77.3	83.0	92.9	
16–18 weeks OGTT blood glucose T0′ (mg/dl)	98.8 ± 9.4	95.9 ± 9.4	92.6 ± 9.0	90.0 ± 9.6	**0.002**
16–18 weeks OGTT blood glucose T60′ (mg/dl)	174.0 ± 34.3	170.1 ± 37.3	179.3 ± 30.2	143.6 ± 35.9	**0.02**
16–18 weeks OGTT blood glucose T120′ (mg/dl)	141.2 ± 35.1	142.5 ± 33.1	143.0 ± 33.0	123.8 ± 30.2	0.16
24–28 weeks OGTT blood glucose T0′ (mg/dl)	79.8 ± 35.6	80.7 ± 31.9	84.5 ± 20.5	82.3 ± 19.3	** < 0.0001**
24–28 weeks OGTT blood glucose T60′ (mg/dl)	179.6 ± 27.5	174.9 ± 30.4	176.1 ± 28.8	176.1 ± 30.5	0.82
24–28 weeks OGTT blood glucose T120′ (mg/dl)	146.9 ± 29.2	149.4 ± 31.5	151.6 ± 33.5	147.1 ± 31.1	0.61
Follow-up OGTT T0′ (mg/dl)	95.6 ± 12.9	92.1 ± 10.1	90.6 ± 8.6	87.6 ± 9.0	** < 0.0001**
Follow-up OGTT T120′ (mg/dl)	110.3 ± 33.1	101.8 ± 30.1	104.4 ± 24.2	95.8 ± 24.7	** < 0.0001**
Systolic blood pressure (mmHg)	111.8 ± 12.4	109.9 ± 13.0	116.7 ± 13.1	109.5 ± 12.0	** < 0.0001**
Diastolic blood pressure (mmHg)	69.1 ± 8.9	68.9 ± 9.2	72.2 ± 9.8	68.3 ± 8.8	**0.002**
Total cholesterol (mg/dl)	249.7 ± 46.3	251.3 ± 53.3	245.7 ± 56.1	243.1 ± 40.4	0.66
HDL cholesterol (mg/dl)	68.7 ± 16.0	70.1 ± 16.1	68.6 ± 16.7	69.5 ± 14.9	0.76
LDL Cholesterol (mg/dl)	143.5 ± 44.0	146.8 ± 50.1	141.5 ± 49.9	140.5 ± 36.2	0.97
Triglycerides (mg/dl)	212.9 ± 81.5	185.5 ± 73.3	186.7 ± 75.7	168.7 ± 89.0	** < 0.0001**
Glucose lowering treatment (%)					
Diet	0	0	100	100	** < 0.0001**
Insulin	100	100	0	0	
Gestational week at delivery	38.2 ± 1.7	38.6 ± 1.2	38.8 ± 1.3	38.9 ± 1.4	** < 0.0001**
Adverse neonatal outcome (%)	37.9	31.0	24.8	23.8	**0.003**

Student’s test (for continuous, normally distributed variables), Mann–Whitney test (for continuous, not normally distributed variables) or Chi-square test (for categorical variables)

*p values < 0.05 are in bold and expresses statistically significant between-group differences

**Fig. 3:**
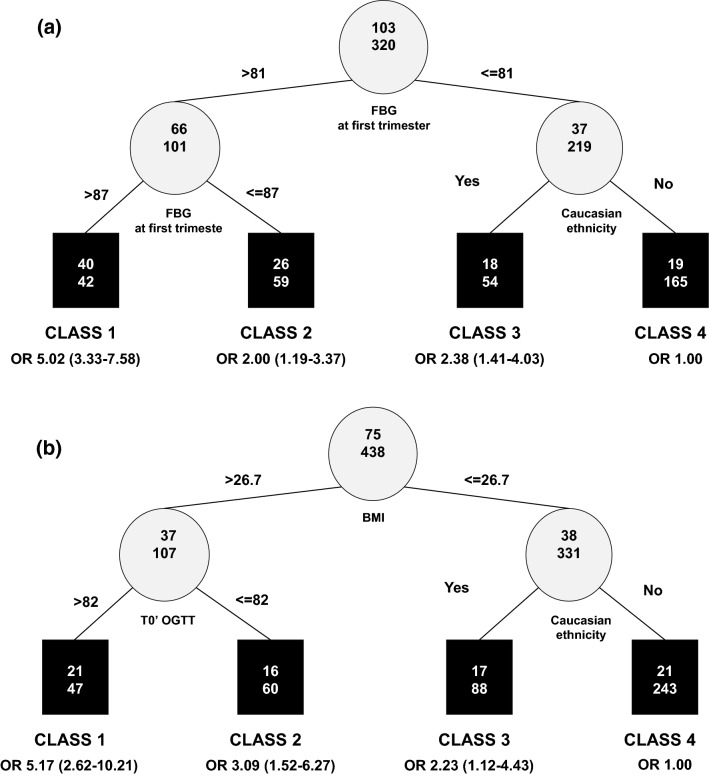
Additional exploratory RECPAM analyses on the subgroups of women with available data on FBG at first trimester and OGTT 24–28 weeks. **a** Identification of subgroups at different risks of developing postpartum dysglycemia including FBG (mg/dl) at first trimester of pregnancy as additional covariate (N = 423). **b** Identification of subgroups at different risks of developing postpartum dysglycemia including OGTT at 24–28 weeks (mg/dl) as additional covariate (N = 513)

Data on fasting BG in the first trimester, OGTT at 16–18 weeks and OGTT at 24–28 weeks were available for 423 women (44.9%), 96 (10.2%) and 514 (54.6%), respectively. Additional exploratory RECPAM analyses in the two subgroups with adequate sample size (the subgroup with OGTT at 16–18 weeks was too small to allow additional analyses) were performed to assess the role of these BG values on the risk of postpartum dysglycemia (Fig. 3). In the two RECPAM models, fasting BG at first trimester represented the first selected splitting variable, while BG at T0′ of OGTT was selected as the second splitting variable after BMI. In both cases, the cut-offs associated with increased risk ranged between 81 and 87 mg/dl, therefore lower than 92 mg/dl, which is the usually accepted threshold for the diagnosis of GDM.

## Discussion

### Main findings

This Italian observational study shows that among women with a recent history of GDM only about one in three is compliant with the recommended follow-up OGTT within 12 weeks, despite the high risk of postpartum dysglycemia related to GDM.

Our study identified a specific phenotype of women more likely to be compliant with postpartum OGTT, i.e., women with more severe GDM (as documented by more frequent use of insulin) and attitude to be adherent to the overall care (as documented by the better control of all parameters). However, our study also clarifies that compliance with follow-up OGTT is largely dependent from organizational aspects of each center, as documented by the different proportions of women who performed it in the different centers.

Furthermore, our study documents that among women with follow-up OGTT, about 1/4 developed postpartum dysglycemia. IFG was the most common glucose metabolism abnormality, being present in 60.8% of the women as the only alteration. RECPAM analyses show that fasting BG more than post-prandial BG deserve particular consideration: fasting values during pregnancy ranging between 81 and 87 mg/dl (4.5 and 4.8 mmol/l), lower than the current diagnostic threshold of 92 mg/dl (5.1 mmol/l) for GDM at T0′, are associated with increased risk of postpartum dysglycemia.

In addition, our study documents that, among a set of known risk factors, insulin treatment (as a proxy of more severe GDM), pre-pregnancy BMI, and family history of diabetes are the key variables for identifying subgroups of women with a from twofold to fivefold higher risk of developing dysglycemia compared to the reference class (women with BMI lower than 28 kg/m^2^ not requiring insulin for GDM).

### Comparison with existing data

In other countries, among women with GDM, postpartum IGT prevalence ranged between 17 and 23% and diabetes prevalence between 5 and 14% [16–18], and rates of postpartum follow-up was low in most parts of the world [17, 18].

Prevalence of postpartum dysglycemia and distribution of IFG/IGT after postpartum varied according to different ethnic populations [19]. It is also known that traditional cardiovascular risk factors (especially high levels of BMI) have a stronger association with isolated IFG than with isolated IGT in women with prior GDM [20].

It is noteworthy that many prominent barriers for non-compliance with follow-up OGTT could be overcome through appropriate educational sessions [21, 22] and reminder/engagement systems [23, 24]. In this respect, previous studies have documented that fasting BG and/or HbA1c are not sensitive enough to replace the OGTT in early postpartum [25].

Recently, Waters et al. documented in women with GDM that no significant correlations were present between longitudinal changes in maternal lipids, body weight, and inflammatory markers and changes in insulin sensitivity, insulin response and disposition index from late pregnancy and during the early postpartum period. Therefore, owing to difficulties in completing an OGTT in the later postpartum period (6–12 weeks), screening within 1–5 days postpartum is proposed as a viable option [24].

Established risk factors for GDM include ethnicity, obesity, and family history of diabetes [26, 27], while lower insulin sensitivity, use of insulin therapy, pre-pregnancy obesity, severity of GDM and high HbA1c levels during pregnancy were identified as independent predictors of subsequent diabetes in previous observational studies [28–30], in line with our data. Our study adds novel information about their interaction and their priority in identifying specific phenotypes. In addition, while the predictive role of first trimester BG values is recognized [31], only another study identified a cut-off of fasting BG lower than 92 mg/dl (5.1 mmol/l) and near to 80 mg/dl (4.4 mmol/l) as a risk factor in this population, although the outcome was GDM and not postpartum dysglycemia, in line with our data [32].

### Implications for research and clinical practice

Increasing the awareness of the risk of dysglycemia after GDM is a priority. Early diagnosis of postpartum dysglycemia provides an opportunity to use dietary, lifestyle, and pharmacological interventions that might prevent or delay the onset of type 2 diabetes in GDM women [33–38]. A return to pre-pregnancy weight within 1 year postpartum should be the goal [39–41].

Timing of follow-up OGTT should be more clearly defined. Some guidelines recommend that after delivery all women with GDM have to be re-evaluated by a 75 g OGTT (WHO criteria) 4–12 weeks postpartum to reclassify the glucose tolerance and every 2 years in cases of normal glucose tolerance, but evidence level is B [42].

The reliability of follow-up OGTT within few days after delivery deserves further investigation [24].

New research is focused on genetic, immunologic and biological markers to predict future GDM or T2DM [43]. However, in clinical practice it remains important to “phenotyping” the women at risk on the basis of easily available information.

### Strengths and limitations

As the main strength, this is a large national multicenter study giving a national picture of the care and the outcome of pregnancies complicated by GDM. Study limitations were the retrospective design, not having planned a longer mother and children follow-up and not having collected information about specific ethnicities of women.

## Conclusions

Increasing compliance to postpartum OGTT to prevent or delay type 2 diabetes onset after GDM is a priority. Easily available patient characteristics such as socioeconomic status, family history of diabetes, previous GDM, pre-pregnancy BMI, need for insulin for GDM treatment, and fasting BG in the first phase of pregnancy at lower thresholds than those currently utilized, can help focusing on those women who are more likely to benefit from preventive strategies.

## Data Availability

The datasets generated during and/or analyzed during the current study are available from the corresponding author on reasonable request.

## Abbreviations

95% CI: 95% Confidence intervals
BG: Blood Glucose
BMI: Body mass index
GCP: Good Clinical Practice
GDM: Gestational diabetes mellitus
IFG: Impaired Fasting Glucose
IGT: Impaired Glucose Tolerance
OGTT: Oral Glucose Tolerance Test
OR: Odds Ratio
RECPAM: RECursive Partitioning and AMalgamation
T0′: OGTT-baseline value
T120′: OGTT-value after 2 h
