# Correction to: Screening of postpartum diabetes in women with gestational diabetes: high‑risk subgroups and areas for improvements—the STRONG observational study

**DOI:** 10.1007/s00592-021-01730-w

**Published:** 2021-06-11

**Authors:** Angela Napoli, Laura Sciacca, Basilio Pintaudi, Andrea Tumminia, Maria Grazia Dalfrà, Camilla Festa, Gloria Formoso, Raffaella Fresa, Giusi Graziano, Cristina Lencioni, Antonio Nicolucci, Maria Chiara Rossi, Elena Succurro, Maria Angela Sculli, Marina Scavini, Ester Vitacolonna, Matteo Bonomo, Elisabetta Torlone, the STRONG Study Collaborators and the AMD-SID Diabetes and Pregnancy Study Group, Angela Napoli, Olimpia Bitterman, Camilla Festa, Chiara Giuliani, Matteo Bonomo, Basilio Pintaudi, Elena Cimino, Elena Mion, Teresa Marcone, Cristina Lencioni, Graziano Di Cianni, Laura Sciacca, Andrea Tumminia, Agostino Milluzzo, Ester Vitacolonna, Federica Fraticelli, Marica Franzago, Alessandro Roberto Dodesini, Elena Ciriello, Mariagrazia Dalfrà, Annunziata Lapolla, Raffaella Fresa, Aurora Grassi, Paolo Limone, Annamaria Nuzzi, Andi Masha, Laura Grimaldi, Sara Biglino, Egle Ansaldi, Maurizia Battezzati, Giancarla Meregalli, Valentina De Mori, Denise Berzi, Antonio Bossi, Viviana Baggi, Elisabetta Lovati, Lara Quarleri, Tiziana Romanelli, Silvia Clementi, Ilaria Nicolao, Francesca Zambotti, Simonetta Lombardi, Silvana Costa, Chiara Tommasi, Silvia Rancan, Giovanna Lisato, Paola Bordon, Daniela Turazzi, Francesco Mollo, Franco Grimaldi, Laura Tonutti, Sandra Agus, Maria Rosaria Falivene, Giorgio Versari, Laura Corsi, Maria Delucchi, Luisa Ratto, Maria Grazia Magotti, Tiziana Frusca, Silvia Haddoub, Alice Suprani, Mary Mori, Maria Grazia Vita, Nicolina Di Biase, Alessandra Bertolotto, Michele Aragona, Cristina Bianchi, Emilia Lacaria, Elisa Guarino, Federica Monaci, Francesco Dotta, Elisabetta Torlone, Carlo Lalli, Chiara Di Loreto, Maura Scarponi, Angela Del Prete, Sergio Leotta, Iolanda Coletta, Santina Abbruzzese, Valeria Montani, Emanuela Cannarsa, Pierpaolo Contini, Raffaella Vero, Rosa Oliverio, Marina Scavini, Nicoletta Dozio, Maria Pia Imbergamo, Renzo Cordera, Laura Affinito, Davide Maggi, Caterina Bordone, Elena Fochesato, Alessandra Pissarelli, Eros Libera, Susanna Morano, Tiziana Filardi, Mara Fallarino

**Affiliations:** 1 AMD-SID Diabetes and Pregnancy Study Group, Rome, Italy; 2 Department of Clinical and Molecular Medicine, Sant’Andrea Hospital, Faculty of Medicine and Psychology, Sapienza University, Rome, Italy; 3 Department of Clinical and Experimental Medicine, Endocrinology Section, University of Catania Medical School, Catania, Italy; 4 SSD Diabetology, Ca’Granda Niguarda Hospital, Milan, Italy; 5 Department of Medicine, University of Padova, Padova, Italy; 6 Department of Medicine and Aging Sciences; Center for Advanced Studies and Technology (CAST, Ex CeSI-Met), G. D’Annunzio University, Chieti, Italy; 7 Endocrinology and Diabetes Unit, ASL Salerno, Salerno, Italy; 8 CORESEARCH – Center for Outcomes Research and Clinical Epidemiology, Pescara, Italy; 9 Diabetes and Endocrinology Unit, Usl Nord Ovest Tuscany, Lucca, Italy; 10 Department of Medical and Surgical Sciences, University Magna Graecia of Catanzaro, Catanzaro, Italy; 11 Endocrinology and Diabetes, Bianchi Melacrino Morelli Hospital, Reggio Calabria, Italy; 12 Division of Immunology, Transplantation and Infectious Diseases, Diabetes Research Institute (DRI), IRCCS San Raffaele Scientific Institute, Milan, Italy; 13 Department of Medicine and Aging, School of Medicine and Health Sciences, “G. D’Annunzio” University, Chieti-Pescara, Chieti, Italy; 14 Internal Medicine, Endocrinology and Metabolism, S. Maria Della Misericordia Hospital, Perugia, Italy

## Correction to: Acta Diabetologica https://doi.org/10.1007/s00592-021-01707-9

Authors would like to correct the error in their publication.

Figure 3 contains a part in Italian language which is now removed.

The collaborator author names were tagged only in the author group but missed to process in Acknowledgements section. The collaborator author names now updated as Study group in the Acknowledgements section.

The original article has been corrected.

